# Screening highly active perovskites for hydrogen-evolving reaction via unifying ionic electronegativity descriptor

**DOI:** 10.1038/s41467-019-11847-w

**Published:** 2019-08-21

**Authors:** Daqin Guan, Jing Zhou, Yu-Cheng Huang, Chung-Li Dong, Jian-Qiang Wang, Wei Zhou, Zongping Shao

**Affiliations:** 1 State Key Laboratory of Materials-Oriented Chemical Engineering, College of Chemical Engineering, Nanjing Tech University, Nanjing, 211800 China; 2 Shanghai Institute of Applied Physics, Chinese Academy of Sciences, Shanghai, 201204 China; 3 Department of Physics, Tamkang University, 151 Yingzhuan Rd., New Taipei City, 25137 Taiwan

**Keywords:** Catalytic mechanisms, Inorganic chemistry, Materials chemistry, Electrocatalysis

## Abstract

Facile and reliable screening of cost-effective, high-performance and scalable electrocatalysts is key for energy conversion technologies such as water splitting. ABO_3-*δ*_ perovskites, with rich constitutions and structures, have never been designed via activity descriptors for critical hydrogen evolution reaction (HER). Here, we apply coordination rationales to introduce A-site ionic electronegativity (AIE) as an efficient unifying descriptor to predict the HER activities of 13 cobalt-based perovskites. Compared with A-site structural or thermodynamic parameter, AIE endows the HER activity with the best volcano trend. (Gd_0.5_La_0.5_)BaCo_2_O_5.5+*δ*_ predicted from an AIE value of ~2.33 exceeds the state-of-the-art Pt/C catalyst in electrode activity and stability. X-ray absorption and computational studies reveal that the peak HER activities at a moderate AIE value of ~2.33 can be associated with the optimal electronic states of active B-sites via inductive effect in perovskite structure (~200 nm depth), including Co valence, Co-O bond covalency, band gap and O 2*p*-band position.

## Abstract (web summary)

Facile and reliable screening of efficient electrocatalysts is important for energy conversion technologies such as water splitting. Here, authors introduce A-site ionic electronegativity as a descriptor to understand the hydrogen-evolution activities of cobalt-based perovskites.

## Introduction

Exploiting fossil-free, renewable and clean energy sources to meet worldwide fuel demand is one crucial challenge facing us today^1^. Hydrogen, as a pivotal feedstock for various chemicals (e.g., methanol and ammonia) in industry, is regarded as the ideal fuel for the future^1,2^. Electrochemical water splitting using renewable electricity as energy input is the most promising technique to achieve sustainable and large-scale hydrogen production^1^. However, efficient and cost-effective hydrogen-evolution reaction (HER) electrocatalysts are highly demanded^1,2^. Although materials containing noble metals (e.g., Pt, Ru, Pd, Rh, Ir) demonstrate favorable HER activities in alkaline solutions^3^, scarcity and high-cost features limit their commercial applications. Considering the tremendous numbers of nonprecious metal-based candidates for HER electrocatalysts, the development of a facile and effective activity descriptor thus becomes extremely helpful in material design and selection.

Earth-abundant, inexpensive and scalable first-row (3*d*) transition-metal-based perovskite oxides, with flexible crystal structure and electronic structure degrees of freedom, can be well tailored for targeting electrochemical reactions^4,5^. To effectively discover or design highly active perovskite candidates for a given reaction, extensive efforts have been devoted to unveiling the relationship between parameter and performance. During the last decade, numerous activity descriptors of perovskites for oxygen electrocatalysis, such as oxygen evolution and reduction reactions (OER and ORR), have been established^4,6–9^. However, no similar achievements for perovskites in alkaline HER have been attained due to their inefficient conversion of hydrogen intermediates to H_2_ for oxides^3^. It is noteworthy that very recent advances in perovskite oxides have shown their promising future in alkaline HER^10–12^. Developing an efficient and accurate descriptor for fast screening perovskite-type HER electrocatalysts thus becomes an urgent scientific issue.

To date, almost all the activity descriptors reported for perovskites were directly based on the physicochemical properties of active sites^4^. In perovskite lattice (ABO_3_; A = rare-earth or alkaline-earth metal cations, B = transition-metal ions), B-sites coordinated with O anions are generally identified as active sites for adsorption and desorption of reaction intermediates^4^. Theoretically, the calculated adsorption strengths of oxygen species (for OER or ORR)^7,13,14^ and hydrogen intermediates (for HER)^15^ on active sites are acknowledged as universal descriptors. Lee^16^, Hong^17^, Calle-Vallejo^18^, Wu^19^, Fung^20,21^, and Govindarajan^22^ et al. also reported the position of the O *p*-band center, the charge-transfer energy, the bulk formation energy, the bond-energy-integrated coordination number, the adjusted coordination number and the electrochemical-step symmetry index as OER/ORR activity descriptors, respectively. Experimentally, *e*_g_ occupancy^6^, B-site oxidation state^23^, outer electrons^24^, multi-physicochemical material properties^25^, and electrochemical redox potentials^26^ were proposed to elucidate the OER/ORR catalytic activities. Despite these successful efforts, key limitations that plague the screening efficiency still remain: time-consuming and high-cost modeling and calculations for band structures or reaction processes are always involved in computational descriptors; and complex high-resolution characterizations such as synchrotron X-ray analytical techniques are indispensable for achieving accurate experimental properties of B-sites in synthesized perovskites for experimental descriptors. As the most important local environments of B-sites, A-sites, however, are often neglected.

In fact, A-site cations can input their effects on the catalytic activity of perovskites in an indirect way by influencing the electronic and chemical states of B-site ions in perovskite lattice. If B-site features can be quantitatively correlated with accessible A-site properties under the identical synthesis conditions, facile screening of HER-active perovskite candidates may be realized via applying A-site properties. Doping different A-site elements into perovskite structure to tune B-site properties actually implies this possibility. Recently, Grimaud^27^, Wang^28^, and Shang^29^ et al. substituted rare-earth lanthanides with different ionic radii on the A-sites to optimize the OER/ORR activities of RBaCo_2_O_6-*δ*_ (R = lanthanides), RNiO_3-*δ*_, and R_2_Ir_2_O_7-*δ*_ systems, respectively. Diaz-Morales^30^ and Retuerto^31^ et al. also proved the effectiveness of A-site tuning strategy in perovskites. In other words, perovskite performance may also be predicted from accessible A-site properties (i.e., ionic radius) under the same preparation conditions. Actually, A-sites coordinated with O anions show various structural (i.e., ionic radius), thermodynamic (i.e., A–O bond energy^32^) and electronic characteristics^33^. As an important electronic property of A-sites, ion electronegativity (defined as the propensity of ions to attract electrons), which involves the structural (ionic radius) and thermodynamic (ionization energy) factors^33,34^, may be a good unifying parameter of choice to reflect B-site properties.

Here, we apply the neighboring configurations of active B-sites to enable fast and rational screening of efficient perovskites for HER, which is different from the methodology of existing descriptors based on B-sites of perovskites. Specifically, by comparing single structural or thermodynamic parameter (such as A-site ionic radius and A–O bond energy), we show that A-site ionic electronegativity (AIE), as a unifying electronic descriptor, has the most predictive power to identify a highly HER-efficient oxide from more than 10 different cobalt-based perovskites (synthesized under the identical conditions) via an optimal volcano-type activity trend. (Gd_0.5_La_0.5_)BaCo_2_O_5.5+*δ*_ (Gd0.5)^10^ predicted from an AIE value of ~2.33 shows a robust HER activity with an extremely high turnover frequency (TOF) value of ~22.9 s^−1^ at overpotential (*η*) of 0.24 V and a very small Tafel slope of 27.6 mV dec^−1^, which is the top-level catalytic performance among all metal oxides ever reported and even outperforms the most active noble metal Pt/C catalyst. Owing to the impact of A-site inductive effects and electron exchange interactions on active B-sites in perovskites under the identical preparation conditions, a moderate AIE value of ~2.33 can be successfully correlated with the optimum Co valence, Co–O bond covalency, band gap and O 2*p*-band position in perovskite lattice (depth ~ 200 nm), as revealed from our X-ray absorption and first-principles results. This work opens up an avenue to accelerate the search for highly active materials utilizing coordination rationales^35,36^, which will help save considerable time and resources for catalyst design and selection.

## Results

### Design of A-site cations in perovskite lattice

**Fig. 1:**
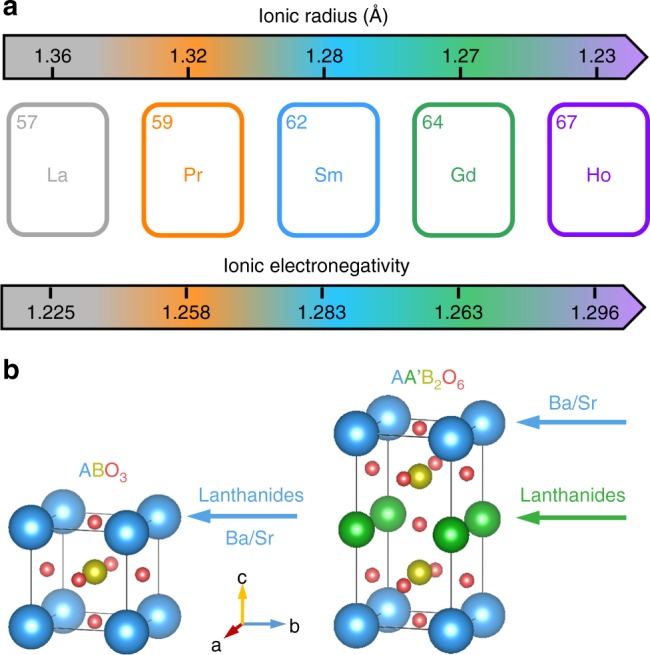
Design of A-sites in perovskite lattice. **a** Values of ionic radius and ionic electronegativity for La^3+^, Pr^3+^, Sm^3+^, Gd^3+^, and Ho^3+^ cations with 12-fold coordination, which exhibit the lanthanide contraction and gadolinium break phenomena, respectively. **b** Doping lanthanides and alkaline-earth metal cations into cobalt-based single perovskites ABO_3_ (B=Co) and A-site ordered double perovskites AA′B_2_O_6_

Cobalt-based heterogeneous catalysts play the most important role in electrochemical energy conversion processes by virtue of the merits of their robustness, abundance, environmental friendliness and accessibility under the environmental atmosphere^5,37^. Here we seek to modulate the A-site ionic radius, A–O bond energy and A-site ionic electronegativity (AIE) in cobalt-based single perovskites ABO_3-*δ*_ and double perovskite family AA′B_2_O_6-*δ*_ (A′ = lanthanides) via doping or mixing lanthanide and alkaline-earth metal ions on A-sites (Fig. 1b). The single perovskites have only one kind of A-site position^6,11^ while the double perovskites possess two typical A-sites (namely A and A′ planes) ordered along *c* axis owing to the large difference of ionic radius between A and A′ cations^10,27,38^ (Fig. 1b). Lanthanide elements (La, Pr, Sm, Gd, and Ho studied here), with the features of lanthanide contraction in ionic radius and gadolinium break in ionic electronegativity^33^, can well regulate the A-site properties in perovskite structure (Fig. 1a). It was observed that doping lanthanides with different ionic radii into the A′ plane could tune the Co valence in double perovskites^27,39^ (under the identical annealing conditions) and further influence the catalytic performance^27^. Theoretically, the unifying ionic electronegativity calculated on the basis of both the ionic radius and ionization energy^33,34^ may be superior to the single factor of the ionic radius in the prediction of catalytic activity. Moreover, we also substituted Sr element in A-sites aimed at investigating the impact of different A-site elements in perovskites (Fig. 1b). By a facile sol–gel method, 13 pure-phase cobalt-based perovskite oxides were successfully synthesized (Supplementary Fig. 1), including LaBaCo_2_O_5.5+*δ*_ (Gd0), (Gd_0.2_La_0.8_)BaCo_2_O_5.5+*δ*_ (Gd0.2), (Gd_0.4_La_0.6_)BaCo_2_O_5.5+*δ*_ (Gd0.4), Gd0.5, (Gd_0.6_La_0.4_)BaCo_2_O_5.5+*δ*_ (Gd0.6), (Gd_0.8_La_0.2_)BaCo_2_O_5.5+*δ*_ (Gd0.8), GdBaCo_2_O_5.5+*δ*_ (Gd1), (Pr_0.5_La_0.5_)BaCo_2_O_5.5+*δ*_ (Pr0.5La0.5), (Sm_0.5_La_0.5_)BaCo_2_O_5.5+*δ*_ (Sm0.5La0.5), (Pr_0.5_Gd_0.5_)BaCo_2_O_5.5+*δ*_ (Pr0.5Gd0.5), Pr(Ba_0.5_Sr_0.5_)Co_2_O_5.5+*δ*_ (PrBa0.5Sr0.5), Ho_0.8_(Ba_0.6_Sr_0.6_)Co_2_O_5.5+*δ*_ (Ho0.8Ba0.6Sr0.6), and (Gd_0.5_La_0.4_)(BaSr_0.1_)Co_2_O_5.5+*δ*_ (Gd0.5La0.4Sr0.1). According to the unique diffraction peaks of perovskites at ~32.5° in the X-ray diffraction (XRD) patterns (Supplementary Fig. 1), our prepared samples can be classified into single and double perovskites, where double perovskites generally show split main peaks^27,39^. This classification will be further clarified in the discussion of structure.

### Correlating with intrinsic HER activity

As the next step, we utilize the A-site ionic radius, A–O bond energy and AIE values to associate with the intrinsic HER activity of our synthesized perovskites (see detailed calculations and values of A-site parameters in Supplementary Note 1 and Supplementary Table 1). In line with the previously reported protocols^10,11^, all overpotentials here are *iR*-corrected and calibrated to the reversible hydrogen electrode (RHE) scale (Supplementary Fig. 2). To assess the intrinsic HER activity^6^, we normalize the HER kinetic current densities to the surface areas of perovskite oxides (defined as *J*_oxide_), which are measured by Brunauer-Emmett-Teller (BET) approach (Supplementary Table 2). Then, the overpotentials required to afford specific current densities of 1 and 10 mA cm^−2^_oxide_ as well as Tafel slopes are compared among 13 prepared perovskites as a function of calculated A-site properties.

**Fig. 2:**
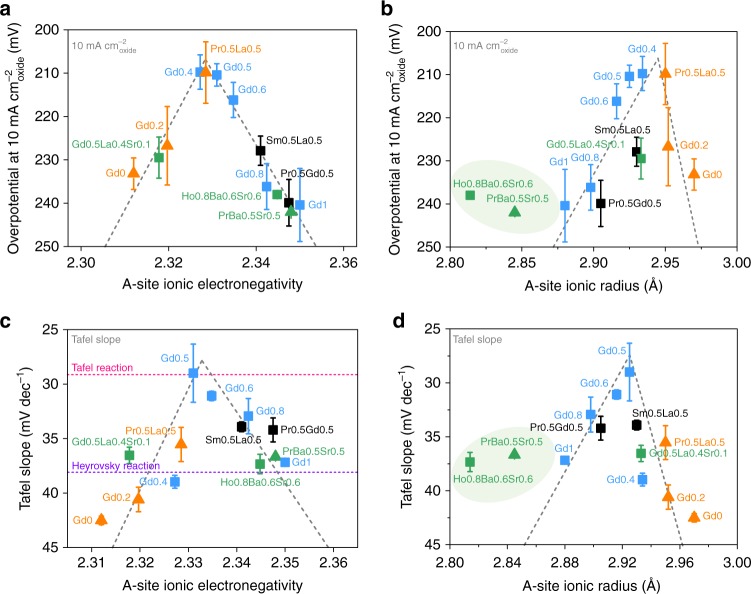
Correlation with intrinsic HER activity. HER activity trends of overpotential at 10 mA cm^−2^_oxide_ as a function of **a** A-site ionic electronegativity, and **b** A-site ionic radius for single and double perovskites studied here. Plots of Tafel slope values as a function of **c** A-site ionic electronegativity (the rate-determining steps estimated from the Tafel slope are shown in pink and purple dashed lines), and **d** A-site ionic radius for the prepared single and double perovskites. Error bars stand for the standard deviation (s.d.) of three independent HER measurements performed in 1.0 M KOH at 25 °C and the gray dashed lines are shown for guidance only. Triangles and squares represent the single and double perovskites, respectively. Sr-doped perovskites (green) and perovskites doped with different lanthanides (Sm0.5La0.5 and Pr0.5Gd0.5 in black) are shown in distinguished colors

We find that the thermodynamic parameter A–O bond energy cannot be rationally correlated with the intrinsic HER activity as no evident activity trends are observed (Supplementary Fig. 3); and the structure factor A-site ionic radius is not applicable to guide the activity of Sr-doped perovskites and the other perovskites roughly represent an imperfect volcano shape (Fig. 2b, d and Supplementary Fig. 4b). As expected, the activity plots based on the unifying AIE parameter show little deviations from the main volcano trends (Fig. 2a, c and Supplementary Fig. 4a), demonstrating that the unifying AIE parameter is more robust and reliable than the single A-site thermodynamic or structure factor in the activity prediction of different perovskites. Our findings here are also supported by the study of Maillard group^40^, where their proposed unifying descriptor is able to address the seemingly contradictory experimental results extracted from the single parameters as well in regard to ORR reactivity. Furthermore, the top HER activity in the volcano schema is found at a moderate AIE value of ≈2.33 (Fig. 2a, c and Supplementary Fig. 4a). Sabatier principle states that the most efficient catalyst should bind the reaction intermediates neither too strongly nor too weakly^1,4^. To fulfill this requirement, moderate parameter magnitudes, such as an *e*_g_ filling close to unity^6^, suitable O 2*p*-band position relative to the Fermi level (*E*_F_)^27,38^ and appropriate valence state^23^, are generally involved in catalyst design to balance the adsorption and desorption strength of reactants. Here we take the view that a moderate AIE value of ≈2.33 may link the optimal active B-sites via some certain effects (under the identical synthetic conditions) and then enable to predict the intrinsic HER activity. If this is valid, the efficiency of screening highly HER-active perovskites will be greatly improved and an enormous amount of time and expenses for catalyst design or selection will be saved through the simple AIE descriptor. Therefore, we further perform structural and electronic configuration characterizations to understand this phenomenon. The AIE design principle here was applied to obtain highly active cobalt-based perovskites for HER. In addition to the intrinsic HER performance, the electrode activities of our prepared perovskites are also investigated and compared with the most prominent catalysts for this reaction in alkaline solutions reported so far.

### Crystal structures and electrode HER activity

**Fig. 3:**
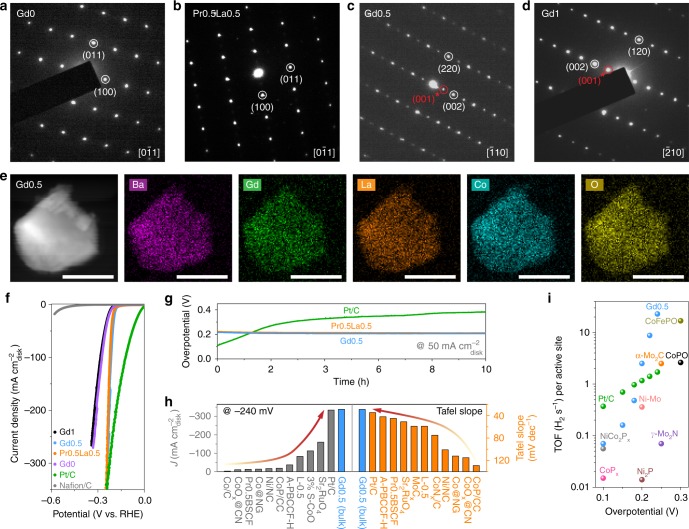
Crystal structure and electrode HER activity. SAED patterns of **a** Gd0, **b** Pr0.5La0.5, **c** Gd0.5, and **d** Gd1 perovskites. **e** HAADF-STEM image and elemental mapping of Gd0.5 sample. Scale bar is 100 nm. **f** Electrode HER polarization curves of Gd1, Gd0.5, Pr0.5La0.5, Gd0, Pt/C, and Nafion/C catalysts. **g** Chronopotentiometric curves of Gd0.5, Pr0.5La0.5, and Pt/C materials at 50 mA cm^−2^_disk_. **h** HER activity comparisons of disk current density at −240 mV and Tafel slope between Gd0.5 perovskite and recently reported state-of-the-art electrocatalysts. **i** Comparisons of TOF values obtained on Gd0.5 material at different overpotentials with those of other well-known HER electrocatalysts

To verify the structure classification mentioned above, combined XRD refinements and high-resolution transmission electron microscopy (HR-TEM) are performed on the representative perovskites (Gd0, Pr0.5La0.5, Gd0.5, and Gd1) in the volcano plots. Cooperative results of XRD refinements and selected-area electron diffraction (SAED) analysis reveal that perovskites such as Gd0 and Pr0.5La0.5, with one single peak at ~32.5° in XRD patterns, adopt a single perovskite structure (*Pm*-3*m* space group) with a lattice parameter of *a* = 3.89 Å and show no superlattice reflections in SAED images^11^ along the [0−11] zone axis (Supplementary Fig. 5a, b, Supplementary Table 3 and Fig. 3a, b). Unlike the single perovskite structure, Gd0.5 and Gd1 which display split main XRD peaks possess at least one doubling lattice parameter (≈7.8 Å) from structural refinement analysis and represent cation-ordering reflections in SAED patterns^27^, demonstrating the typical A-site ordered double perovskite structure (Supplementary Fig. 5c, d, Supplementary Table 3 and Fig. 3c, d). Hence, the structure classification of our synthesized perovskites is valid, which can be simply deduced from the main XRD peaks. Also, this indicates that the unifying AIE descriptor is applicable for both single and double perovskites with various chemical compositions. The scan electron microscopy (SEM) pattern, high-angle annular dark-field scanning transmission electron microscopy (HAADF-STEM) image and elemental mapping of Gd0.5 sample confirm the bulk nature with a particle size of ~ 200 nm and the homogeneously dispersed elements (Supplementary Fig. 6 and Fig. 3e).

To compare with the reported highly active materials for HER in alkaline media, electrode performance of the best single and double perovskites (Pr0.5La0.5 and Gd0.5) in our volcano schema is also evaluated. Large-scale industrial applications of hydrogen production from alkaline electrolyzers always require high current densities (≥200 mA cm^−2^)^41^, therefore, the maximum current densities of our samples are investigated on the electrodes (defined as *J*_disk_) first. Remarkably, the HER rate of Gd0.5 rises rapidly with decreasing negative potential and reaches a current density of 338 mA cm^−2^_disk_ at only −240 mV, which is even superior to the state-of-the-art Pt/C catalyst and all pure-phase metal oxides ever reported (Fig. 3f, h and Supplementary Table 4). As see from Fig. 3h and Supplementary Table 4, the current density of Gd0.5 at −240 mV is ~2.1, ~3.0, and ~112.7 times larger than that of Sr_2_RuO_4_^42^, 3% strained CoO^43^, and Co/C^44^ materials, respectively. The extremely fast HER kinetics of Gd0.5 also induce a small Tafel slope of 27.6 mV dec^−1^, which is the lowest record in non-noble-metal electrocatalysts for alkaline HER to date (Supplementary Fig. 7 and Supplementary Table 4). As a critical metric for gas-evolving electrochemical reactions, TOF, defined as the number of gas molecules evolved per active site per second^45^, is also estimated (detailed calculations in Supplementary Note 2 and Supplementary Fig. 8). Notably, Gd0.5 exhibits an outstanding TOF value of ~ 22.9 H_2_ s^−1^ at overpotential of 240 mV, which is over one order of magnitude higher than the state-of-the-art Pt/C catalyst (~1.7 H_2_ s^−1^ at 240 mV overpotential) and even larger than the TOF value of reported CoFePO^46^ material at 300 mV overpotential (~16.9 H_2_ s^−1^) (Fig. 3i and Supplementary Table 5). For the most efficient single perovskite Pr0.5La0.5 from the volcano plots, it also shows a robust electrode activity (Fig. 3f and Supplementary Table 4), corroborating the universal predictive power of the unifying AIE descriptor for both single and double perovskites. Aside from the activity, operation stability is another important criterion to estimate the practical value of a catalyst. The operating overpotentials of Gd0.5 and Pr0.5La0.5 at 50 mA cm^−2^_disk_ almost remain constant while the overpotential for Pt/C increases distinctly^11,42^ during the chronopotentiometric measurement (Fig. 3g), demonstrating the excellent durability of our perovskites (detailed explanations were given in Supplementary Note 3). The above comparisons of the electrode performance reveal that the single and double perovskites predicted from the AIE descriptor can attain outstanding electrode catalytic behaviors as well, even superior to the most prominent reported catalysts in some aspects.

### Associating with the optimal electronic structure

**Fig. 4:**
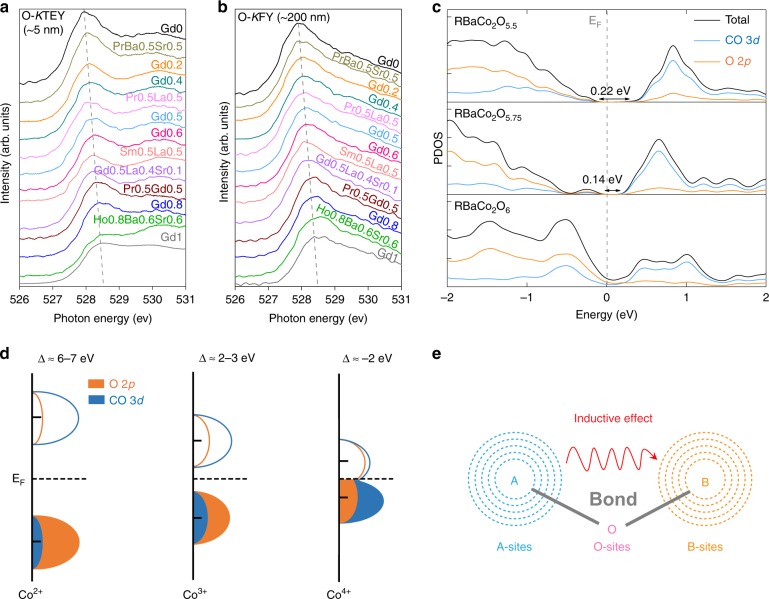
Association with electronic structures. Soft XAS spectra at O-*K* edge collected in **a** TEY and **b** FY modes for all perovskites studied here. **c** PDOS calculations of Co 3*d* and O 2*p* orbitals for Co^3+^ (RBaCo_2_O_5.5_), Co^3.25+^ (RBaCo_2_O_5.75_), and Co^3.5+^ (RBaCo_2_O_6_). **d** Charge-transfer models of Co^2+^, Co^3+^, and Co^4+^ in covalent systems. **e** Inductive effects and electron exchange interactions between A-sites and B-sites in perovskites from molecular orbital theory

To investigate whether the moderate AIE value of ~2.33 can be associated with the ideal electronic configurations (i.e., Co valence, Co–O bond covalency, band gap, and O 2*p*-band position) to fulfill the requirement of Sabatier’s principle, we resort to X-ray adsorption techniques and density functional theory (DFT) calculations. Soft X-ray absorption spectroscopy (XAS) spectra at the O-*K* edge collected by different detection modes are potent to probe the transition-metal valence and metal-oxygen bond covalency in oxides from different depths^10,47^, where the total electron yield (TEY) and fluorescence yield (FY) modes are sensitive to the surface (~5 nm) and bulk (~200 nm) information, respectively. With the increase of Co valence from Co^2+^ (CoO) to Co^3+^ (LaCoO_3_) and further to Co^4+^ (SrCoO_3_), the O-*K* pre-edge peak shifts to lower energies and the spectral weight increases, reflecting the enhanced Co–O bond covalency^10^ (Supplementary Fig. 9). As expected, a neither too low nor too high O-*K* pre-edge energy position and a medium level of spectral weight are observed for Gd0.5 with an AIE value of ~2.33 among all perovskites studied here (Fig. 4a, b), demonstrating its moderate Co valence and Co–O bond covalency. We also find that all our perovskites contain Co^3+^ and Co^4+^ ions and the Co valence of them ranges from ~3.1+ to ~3.4+ when comparing the O-*K* XAS spectra of Co^3+^ and Co^4+^ reference samples (Supplementary Fig. 9). To examine the electronic band structures of different contents of Co^3+^ and Co^4+^ cations in perovskites, the partial density of states (PDOS) calculations of Co 3*d* and O 2*p* orbitals for Co^3+^ (RBaCo_2_O_5.5_), Co^3.25+^ (RBaCo_2_O_5.75_), and Co^3.5+^ (RBaCo_2_O_6_) are performed. With increasing Co valence from Co^3+^ to Co^3.25+^ and further to Co^3.5+^, the Co 3*d* and O 2*p* orbital states predominantly shift close to and even across the *E*_F_, leading to a reduced band gap from 0.22 to 0.14 eV and further to 0 eV (Fig. 4c). These observations are well consistent with the transport property changes from charge-transfer insulator for Co^3+^ to metallic states for Co^4+^ in perovskites as reported^10,48,49^. Considering that the O 2*p*-band center has been proposed as the OER activity descriptor^27,38^, we also evaluate its efficiency in HER here. Notably, the best one Gd0.5 with a moderate AIE value of ~2.33 (Co valence of ~3.25+) shows the O 2*p*-band center neither too close nor too far from the *E*_F_ in Fig. 4c (−2.74, −2.38, and −2.01 eV relative to the *E*_F_ for Co^3+^, Co^3.25+^, and Co^3.5+^, respectively), which is in line with the findings of previous OER works^27,38^. The above analysis of XAS spectra and PDOS calculations can be further supported by the well-proven charge-transfer models^10,50,51^ (Fig. 4d). Generally, an increase of the Co valence from Co^2+^ to Co^4+^ causes a decrease in the charge-transfer energy (∆) from positive (6–7 eV for Co^2+^) to negative (≈ −2 eV for Co^4+^)^10,51^, which is accompanied by the enhanced Co 3*d*-O 2*p* covalency^10,50,51^ (the covalent mixing above and below the *E*_F_ becomes stronger as shown in Fig. 4d) and a shift of the electronic band states closer to the *E*_F_ as well as a narrower band gap. Our prior study^10^ has demonstrated that the oxygen vacancies and the Co^4+^ sites can serve as active sites to adsorb H_2_O and release H_2_ in alkaline HER, respectively, where appropriate numbers of the two active sites in a perovskite with a moderate Co valence (3.25+) can realize a near-optimum synergistic effect to meet the Sabatier principle. If the Co valence is too high (over 3.25+), the oxygen vacancies in perovskites decrease and the insufficient oxygen vacancies can introduce electrochemical barriers for H_2_O adsorption. In the case of perovskites with too low Co valence (below 3.25+), small numbers of Co^4+^ sites are not favorable to desorb H_2_. Theoretically and experimentally, a moderate AIE value of ~2.33 in perovskites here is indeed correlated with the optimal states of active sites to fulfill the fundamental requirement of Sabatier’s principle.

From the standpoints of molecular orbital theory, we seek to understand the origin of the predictive power of the unifying AIE descriptor. In perovskite lattice, A-sites are bridged with B-sites via oxygen atoms, functioning as an important B-site environment (Fig. 4e and Supplementary Fig. 10). The overlap of the electron clouds between A-sites and O-sites deforms the electron cloud of B-sites and further influences B-site properties^52^ under the identical preparation conditions (Fig. 4e and Supplementary Fig. 10). Through such inductive effects^26^ and electron exchange interactions^52^ between A-sites and B-sites (Fig. 4e and Supplementary Fig. 10), B-site electronic structure information thus can be well stored and reflected by the coordinated A-sites. The unifying AIE parameter calculated from structural and thermodynamic factors^33,34^ is the best one to quantitatively describe these electron interactions. Therefore, by using the unifying AIE descriptor among single and double cobalt-based perovskites synthesized under the identical conditions, facile and reliable screening of highly active candidates for HER can be realized.

Following our AIE design principle, we successfully synthesized two perovskite candidates with an AIE value of ~2.33 for the alkaline HER (namely Ba_0.4_Ca_0.6_Gd_0.4_La_0.6_Co_2_O_5.5+*δ*_ and Ba_0.5_Ca_0.5_Pr_0.5_La_0.5_Co_2_O_5.5+*δ*_ as shown in Supplementary Fig. 11a and Supplementary Note 4). As expected, the intrinsic activity of the synthesized perovskites can also climb to the top of the volcano plot (Supplementary Fig. 11b) and the electrode activity is comparable to Gd0.5 (Supplementary Fig. 11c). We also found that the electronic states of the two perovskites are similar to those of Gd0.5 (Supplementary Fig. 11d, e), demonstrating the high efficiency and reliability of our descriptor.

## Discussion

In summary, our methodology of applying the interaction rationales between active sites and their coordinated environments has addressed the challenge of inefficient prediction of highly active materials in the electrochemical field, which will help save considerable time and expenses for catalyst design and selection. We showcase the unifying AIE descriptor which outperforms the single A-site structural or thermodynamic parameter in the efficient screening of high-performance single and double perovskites synthesized under the identical conditions for alkaline HER, where the best one exhibits an extraordinary HER activity with a top-level catalytic TOF value of ~22.9 s^−1^ at overpotential of 0.24 V and a record-breaking Tafel slope of 27.6 mV dec^−1^ among the state-of-the-art materials for this reaction ever reported. Combined XAS and DFT studies demonstrate that the best one with a moderate AIE value of ~2.33 is successfully correlated with the optimal electronic states of active B-sites in perovskites to fulfill the Sabatier’s principle via the inductive effects and electron exchange interactions between A-sites and B-sites from molecular orbital theory. This study opens up an avenue to develop an efficient activity descriptor and provides different insights into the ambient environments of active sites in materials.

## Methods

### Catalyst synthesis

Single and double perovskites studied here were synthesized using a combined ethylenediaminetetraacetic acid-citric acid (EDTA-CA) complexing sol–gel process^11^. Taking the synthesis of (Gd_0.5_La_0.5_)BaCo_2_O_5.5+*δ*_ (Gd0.5) as an example: stoichiometric amounts of Ba(NO_3_)_2_, Gd(NO_3_)_3_•6H_2_O, La(NO_3_)_3_•6H_2_O, and Co(NO_3_)_2_•6H_2_O were dissolved in deionized water. Ethylenediaminetetraacetic acid (EDTA) and citric acid (CA) were then added as complexing agents in sequence at a mole ratio of 1:1:2 for total metal ions/EDTA/CA. To ensure the complete complexation, the pH of the solution was adjusted to 6–7 by the addition of an NH_3_ aqueous solution. A transparent gel was obtained by heating at ~90 °C under stirring. Then, the transparent gel from this method was heated in air at 250 °C for 5 h to obtain a solid precursor. Finally, the solid precursor of perovskites (except Gd0 and Gd0.5La0.4Sr0.1) was further calcined in air at 1000 °C for 10 h to form perovskite powders, where the calcination of the solid precursor for Gd0 and Gd0.5La0.4Sr0.1 was conducted at 1100 °C for 10 h in air to achieve the pure-phase structure. The 20 wt% Pt/C catalyst used in this study was purchased from the company of Johnson Matthey. Analytical grade metal nitrates and other chemicals were purchased from Aladdin Industrial Corporation or Sinopharm Chemical Reagent Co., Ltd., and used as received without further purification.

### Electrochemical measurements

The HER measurements in Ar-saturated 1.0 M KOH solutions were performed at 25 °C on a CHI 760E electrochemistry workstation with a rotating disk electrode (RDE) configuration via using a standard three-electrode electrochemical cell. Catalysts dropped on a 0.196 cm^2^ glassy carbon (GC) electrode (Pine Research Instrumentation), Ag/AgCl with a double junction, and graphite rod were used as the working electrode, reference electrode and counter electrode, respectively. The catalyst inks and working electrodes for HER tests were then prepared^11^. Briefly, perovskite powders (10 mg), carbon black (Super P Li; 10 mg), and 5 wt% Nafion solution (0.1 mL) were dispered in absolute ethanol (1 mL) via mild sonication to produce a homogeneous catalyst ink. Then, 5 μL catalyst ink was parpared onto the GC electrode. For Pt/C electrodes, Pt/C (5 mg) and Nafion solution (0.1 mL) were dispersed in 1 mL of absolute ethanol and an 8.6 μL of the Pt/C ink was transferred onto the GC substrate, which is in line with the literature^11^. All HER potentials here were calibrated with respect to RHE scale (1.002 V vs. Ag/AgCl). The calibration was performed in a H_2_-saturated electrolyte (1.0 M KOH) with a Pt rotating disk electrode (Pine Research Instrumentation) as the working electrode, a graphite rod as the counter electrode, and the double junction Ag/AgCl as the reference electrode. Cyclic voltammetry (CV) was performed and the average of the two potentials at which the current crossed zero, which was −1.002 V vs. Ag/AgCl. This value was taken to be the thermodynamic potential for the hydrogen electrode reaction (Supplementary Fig. 2). The traditional RDE measurements for HER were then conducted. Before each measurement, the system was deaerated by Ar gas for over 30 min. Then, CV scans were performed at a scan rate of 100 mV s^−1^ between −0.9 and −1.6 V vs. Ag/AgCl until the CV curves were reproducible. Subsequently, linear sweep voltammetry (LSV) at a scan rate of 5 mV s^−1^ was conducted. Electrochemical impedance spectra (EIS) were collected at an overpotential of 200 mV ranging from 100 kHz to 0.1 Hz.

### Characterization

XRD patterns of the perovskite powders were analyzed by using filtered Cu-Kα radiation (*λ* = 1.5418 Å) on an X-ray diffractometer (Rigaku Smartlab). Detailed structural information was extracted from the XRD refinements. SAED patterns were collected at 200 kV on a TEM instrument (JEM-2100 UHR, JEOL, Japan). HAADF-STEM image and corresponding elemental mapping were taken at 300 kV on a Tecnai G2 F30 S-Twin TEM (FEI, America). SEM image was performed on a Hitachi S-4800 scanning electron micro-analyzer. BET method (Quantachrome Autosorb-iQ_3_) was applied to obtain the specific surface areas of perovskite powders. Soft XAS spectra at O-*K* edge in TEY and FY modes were conducted at the TLS BL20A of the NSRRC in Taiwan. To calibrate the photon energy, a NiO sample was measured simultaneously for O-*K* edge.

### Theoretical calculations

DFT calculations were performed using the Vienna Ab-initio Simulation Package (VASP) with the projector augmented wave (PAW) method and the Perdew-Burke-Ernzenhof (PBE) functional^53–55^. The Hubbard *U* correction (*U*_eff_ = 3.5 eV) was applied to describe the Co 3*d* electrons in line with prior work^13^. The electronic wave functions were modeled by using a plane-wave basis set with a 600 eV energy cutoff. The electronic structure iteration and the convergence criteria for the geometry optimization were set to 10^−6^ eV and 0.02 eV Å^−1^, respectively. The calculations were sampled in a Monkhorst-Pack 3 × 3 × 3 *k*-point mesh. The O 2*p*-band center was obtained from VASP by taking the centroid of the PDOS of O 2*p* states relative to the Fermi level.

## Supplementary information

Supplementary Information

Source Data

## Data Availability

All relevant data are available from the corresponding authors on request. The source data underlying Figs. 2a–d, 3f–i, 4a-c and Supplementary Figs 1–5, 7–9, and 11 are provided as a Source Data file.
