# Supplementary material for: Screening highly active perovskites for hydrogen-evolving reaction via unifying ionic electronegativity descriptor
Source: Nat Commun. 2019 Aug 21;10:3755. doi: 10.1038/s41467-019-11847-w (PMC6704169; doi:10.1038/s41467-019-11847-w)
Supplement: Supplementary file 1 — Supplementary Information [file 41467_2019_11847_MOESM1_ESM.pdf]

Screening highly active perovskites for hydrogen-evolving reaction via unifying ionic electronegativity descriptor

Guan *et al.*

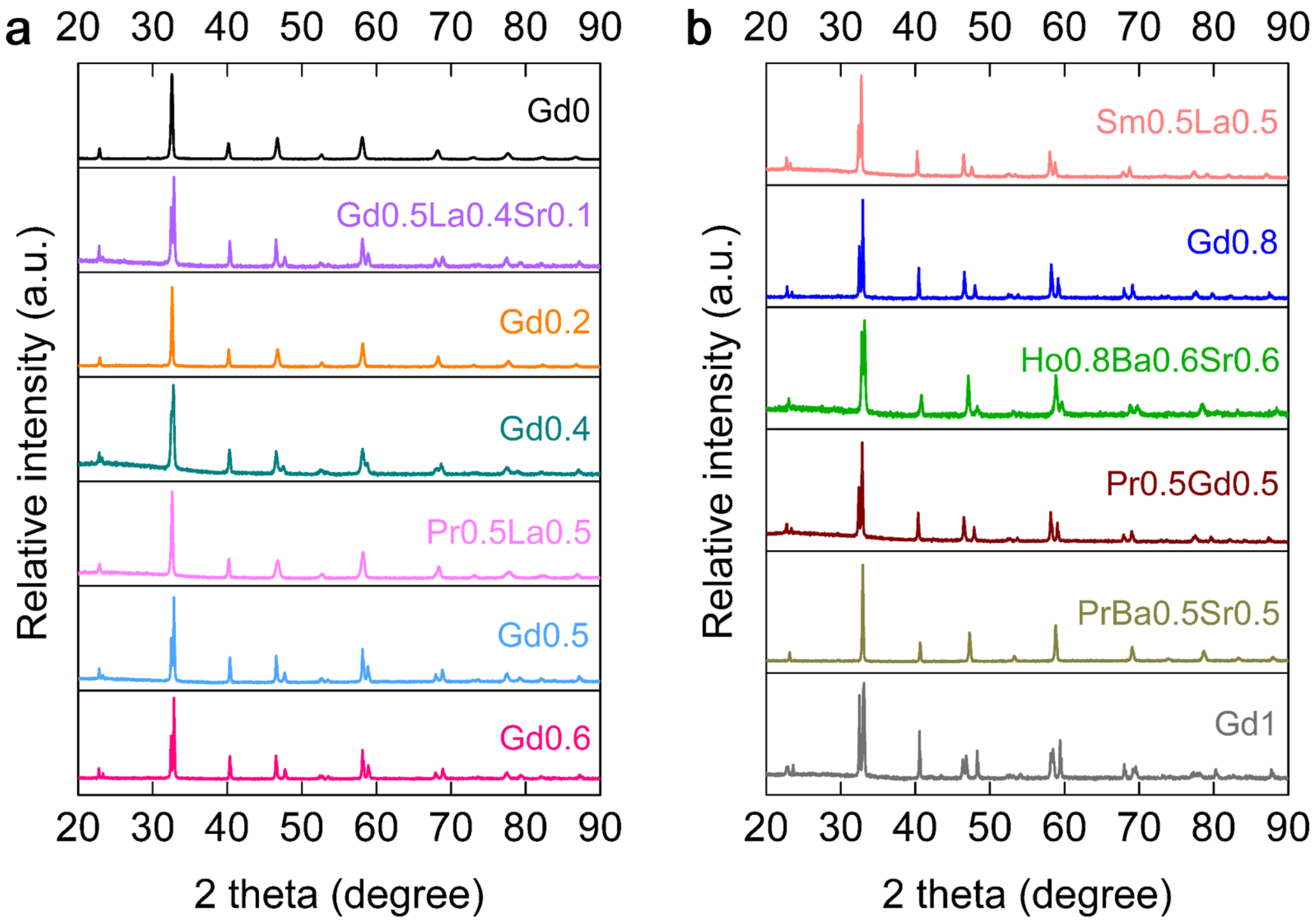

Supplementary Figure 1. XRD patterns of **a,** Gd0, Gd0.5La0.4Sr0.1, Gd0.2, Gd0.4, Pr0.5La0.5, Gd0.5, Gd0.6, **b,** Sm0.5La0.5, Gd0.8, Ho0.8Ba0.6Sr0.6, Pr0.5Gd0.5, PrBa0.5Sr0.5 and Gd1 perovskites, where double perovskites show split main peaks at ~32.5 °.

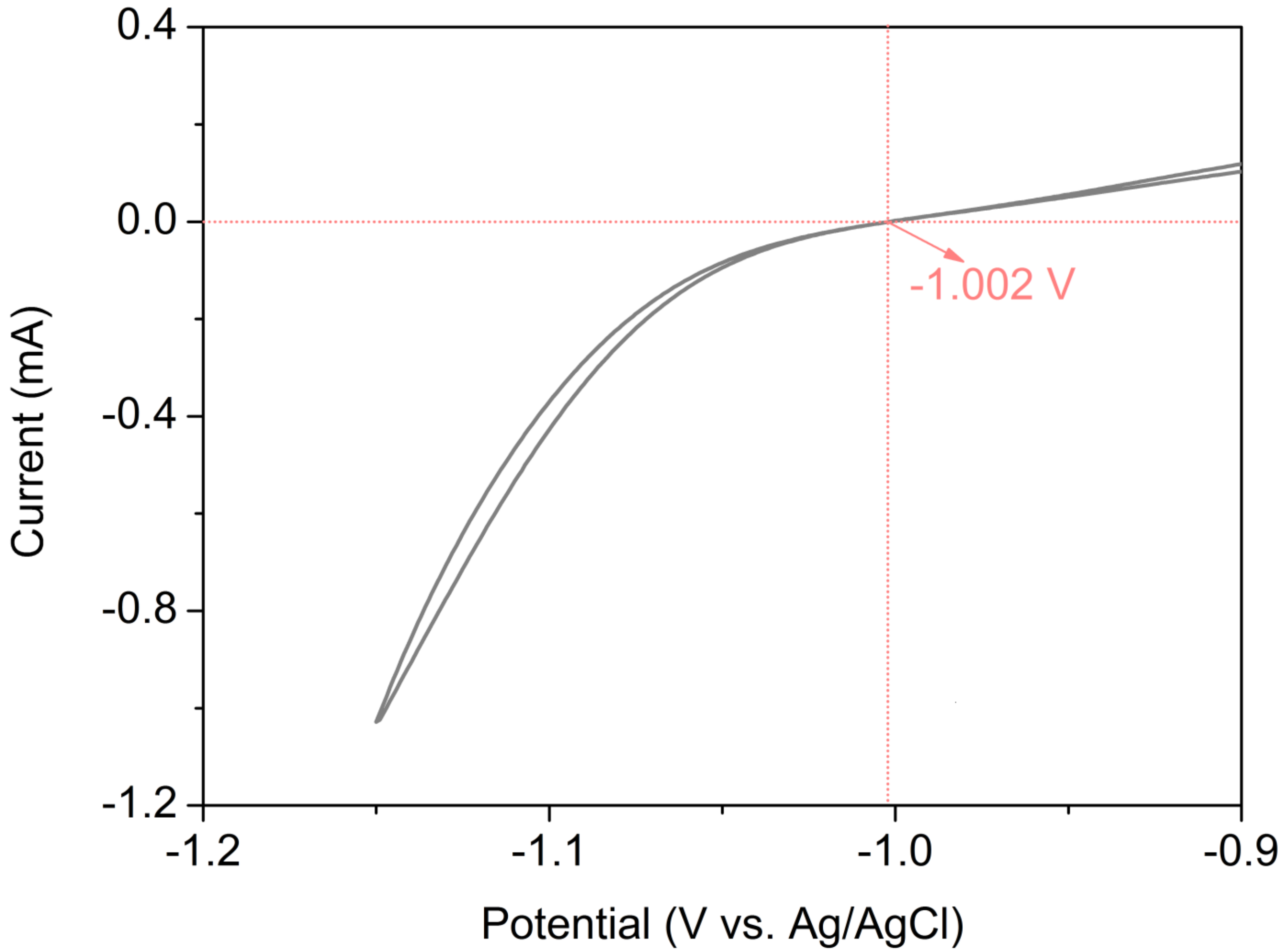

Supplementary Figure 2. Potential calibration of the Ag/AgCl reference electrode at 25 ℃ in 1 M KOH solutions according to precious reported protocols[1].

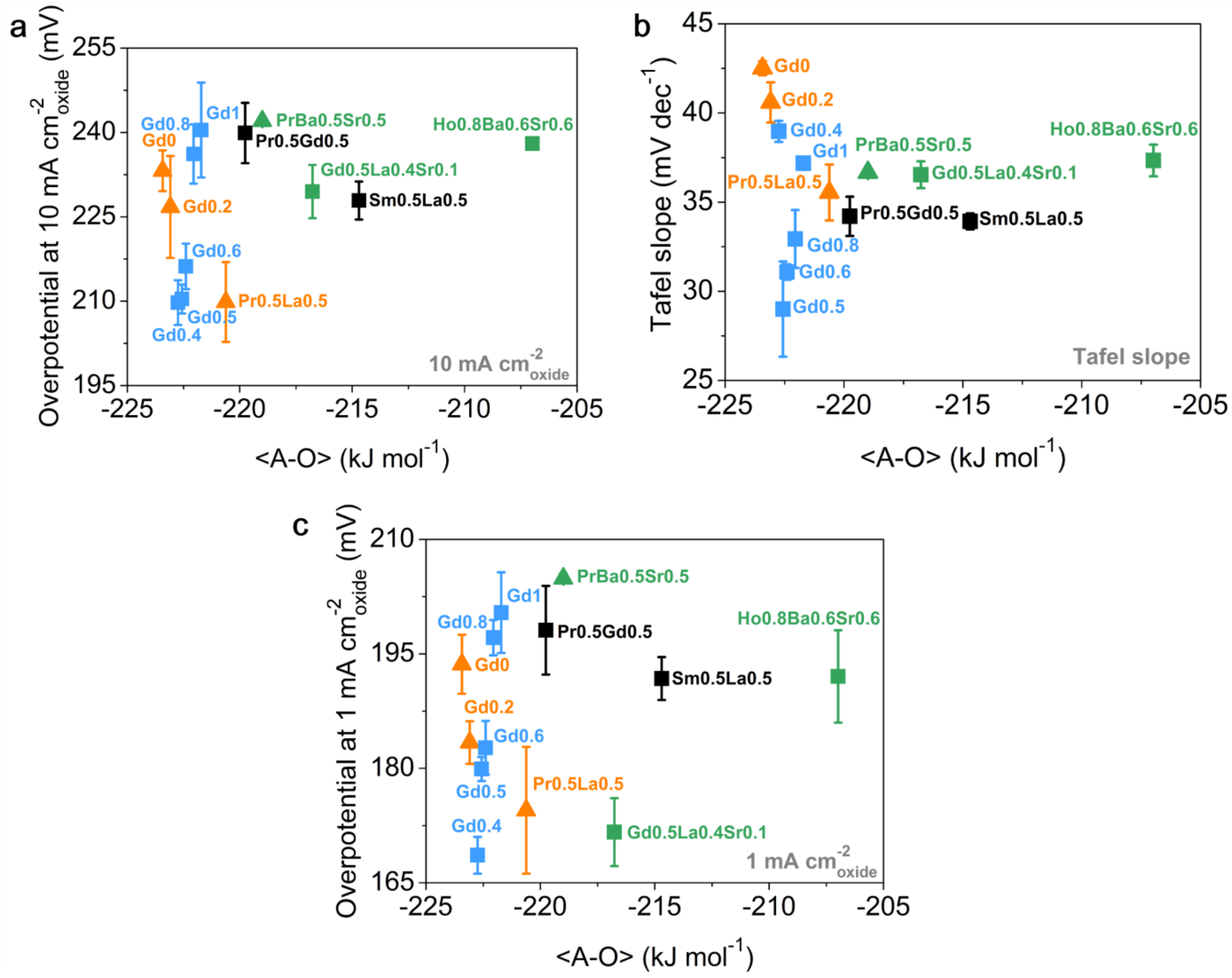

Supplementary Figure 3. Intrinsic HER activity of **a,** overpotential at 10 mA cm$^{-2}_{oxide}$, **b,** Tafel slope and **c,** overpotential at 1 mA cm$^{-2}_{oxide}$ as a function of A-site bond energy for all perovskites studied here. Error bars stand for the standard deviation (s.d.) of three independent HER measurements performed in 1.0 M KOH at 25 ℃.

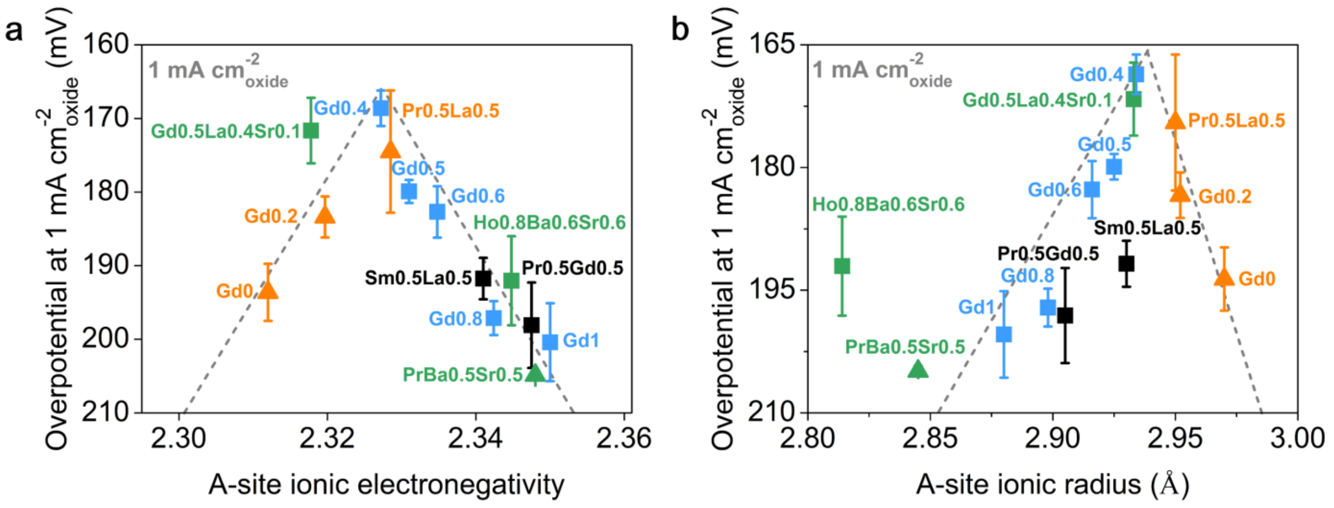

Supplementary Figure 4. Intrinsic HER activity trends of overpotential at 1 mA cm$^{-2}_{oxide}$ as a function of **a,** A-site ionic electronegativity and **b,** A-site ionic radius for single and double perovskites studied here. Error bars stand for the s.d. of three independent HER measurements performed in 1.0 M KOH at 25 ℃ and the grey dashed lines are shown for guidance only.

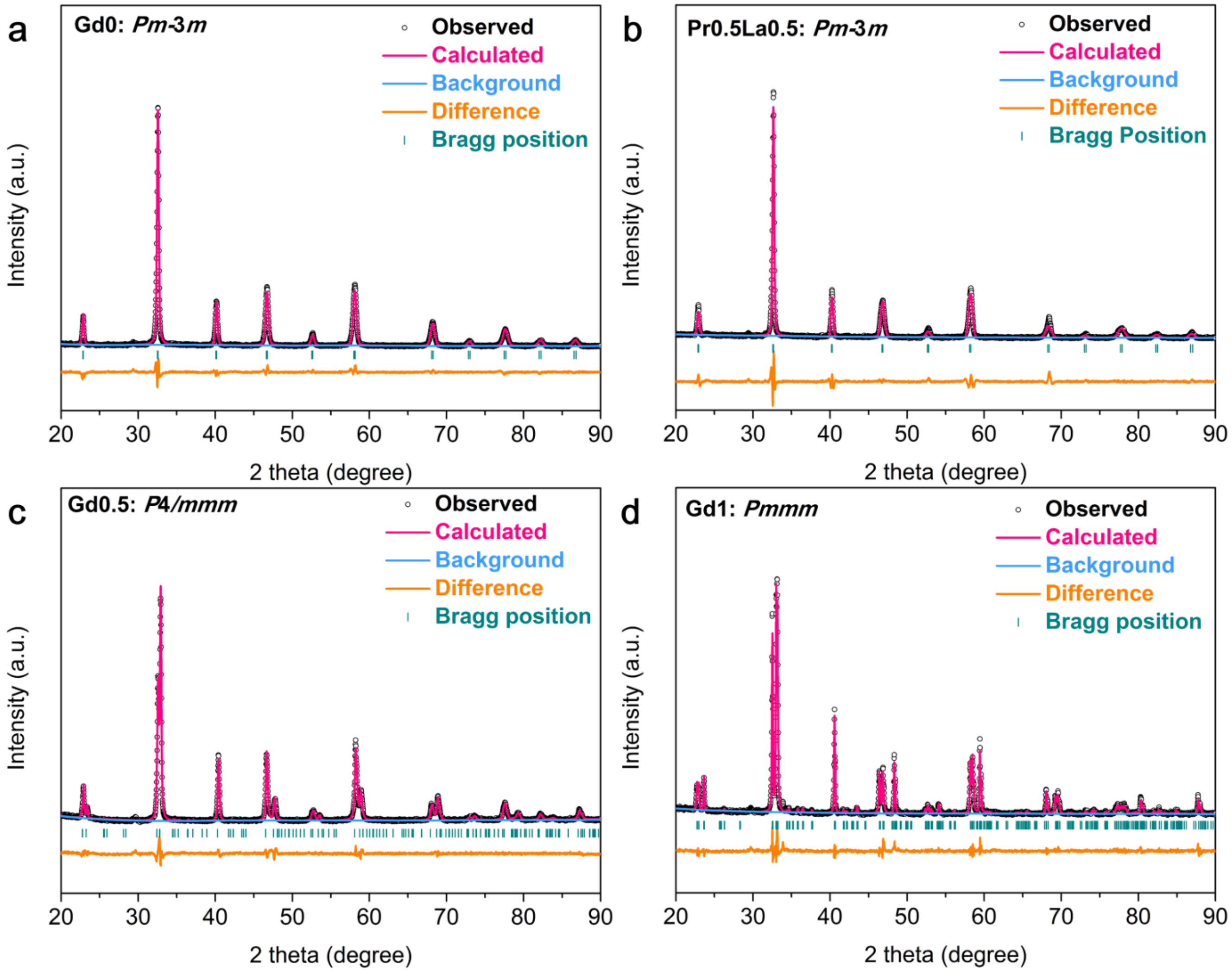

Supplementary Figure 5. Refined XRD profiles of the **a,** Gd0, **b,** Pr0.5La0.5, **c,** Gd0.5 and **d,** Gd1 perovskites.

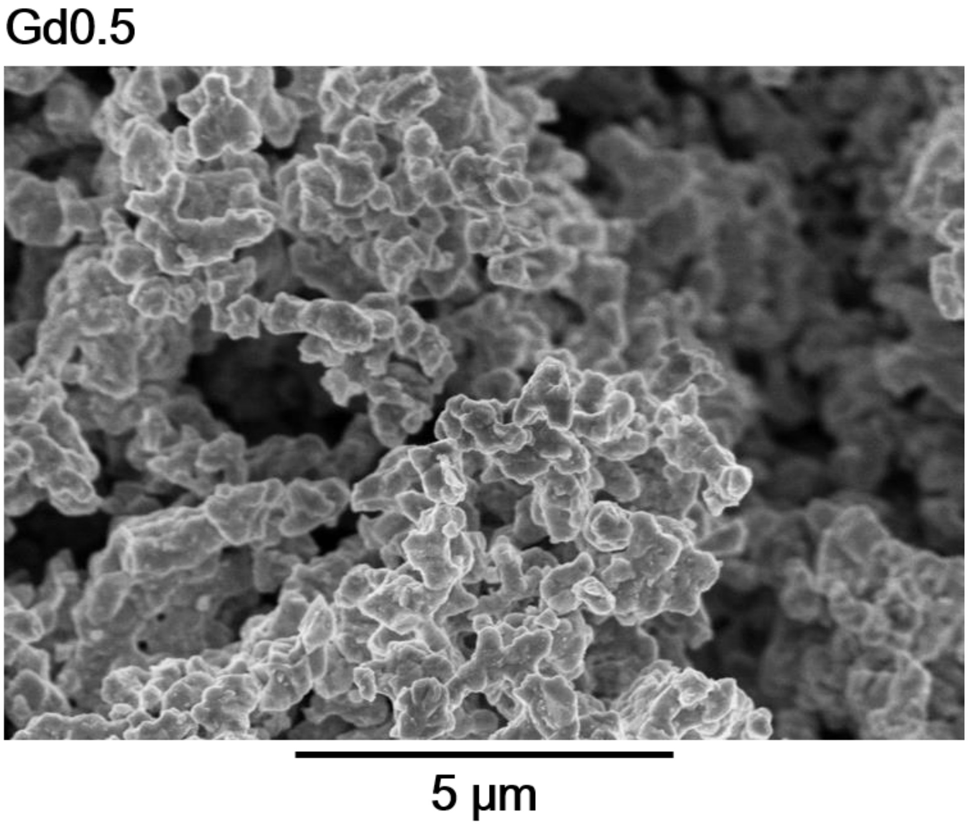

Supplementary Figure 6. SEM image of bulk Gd0.5 sample. Scale bar is 5 μm.

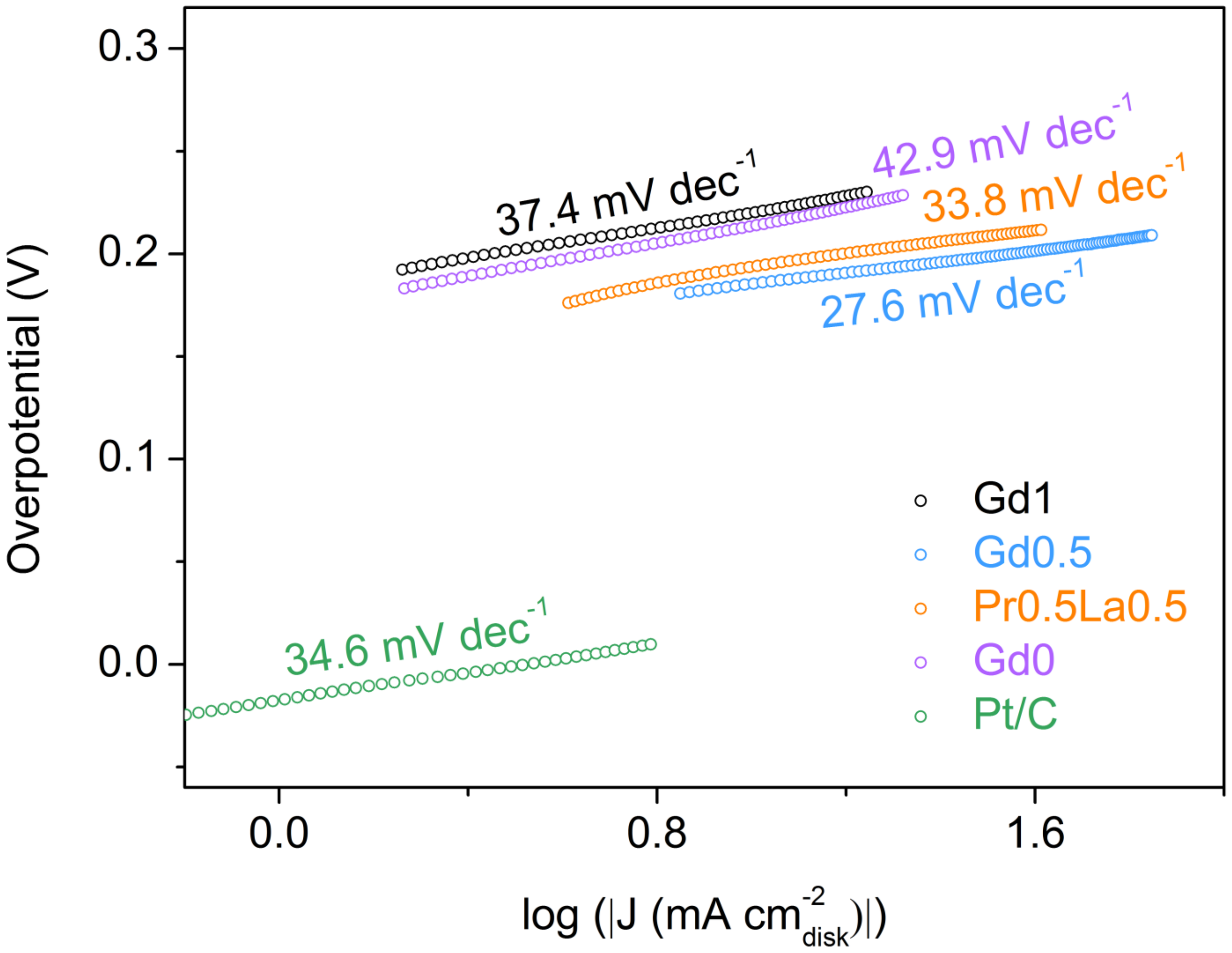

Supplementary Figure 7. Tafel slope of Gd1, Gd0.5, Pr0.5La0.5, Gd0 and Pt/C catalysts.

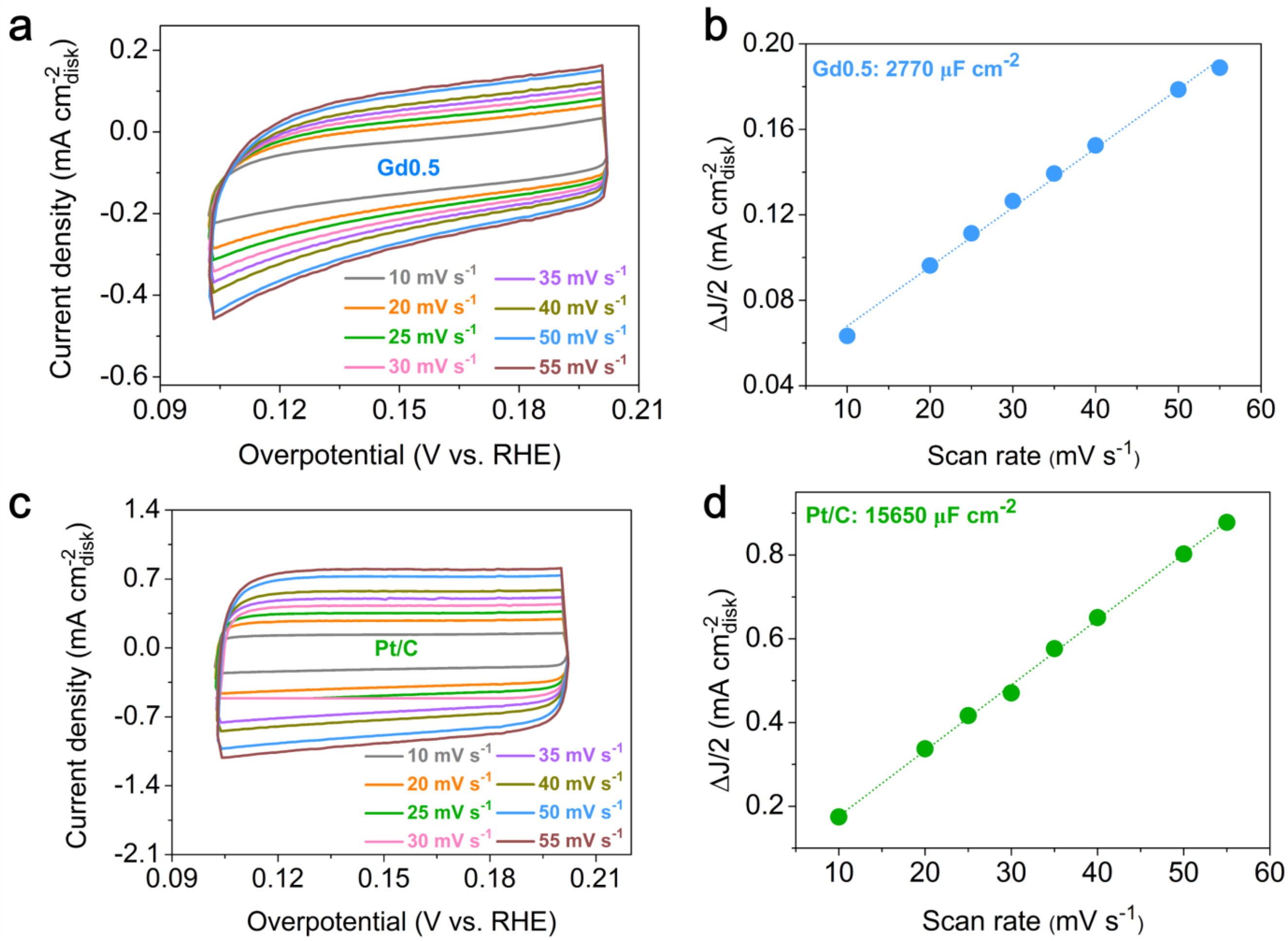

Supplementary Figure 8. Electrochemical cyclic voltammetry scans recorded for **a,** Gd0.5 and **c,** Pt/C materials. Scan rates are 10, 20, 25, 30, 35, 40, 50 and 55 mV s^{-1}. Linear fitting of the capacitive currents versus cyclic voltammetry scans for **b,** Gd0.5 and **d,** Pt/C.

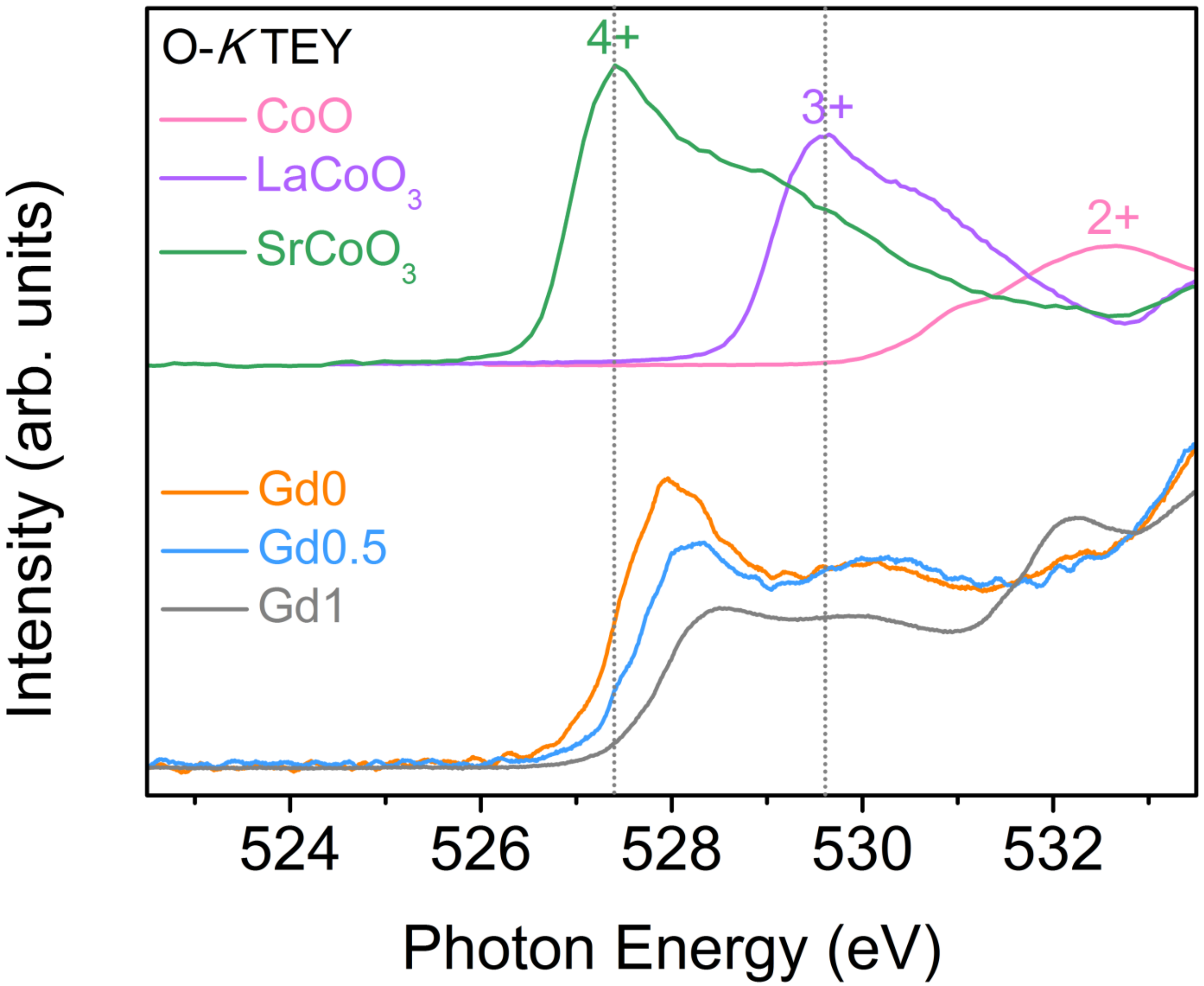

Supplementary Figure 9. The O-*K* XAS spectra in TEY mode for CoO, $LaCoO_3$, $SrCoO_3$ references (top) and samples studied here (bottom).

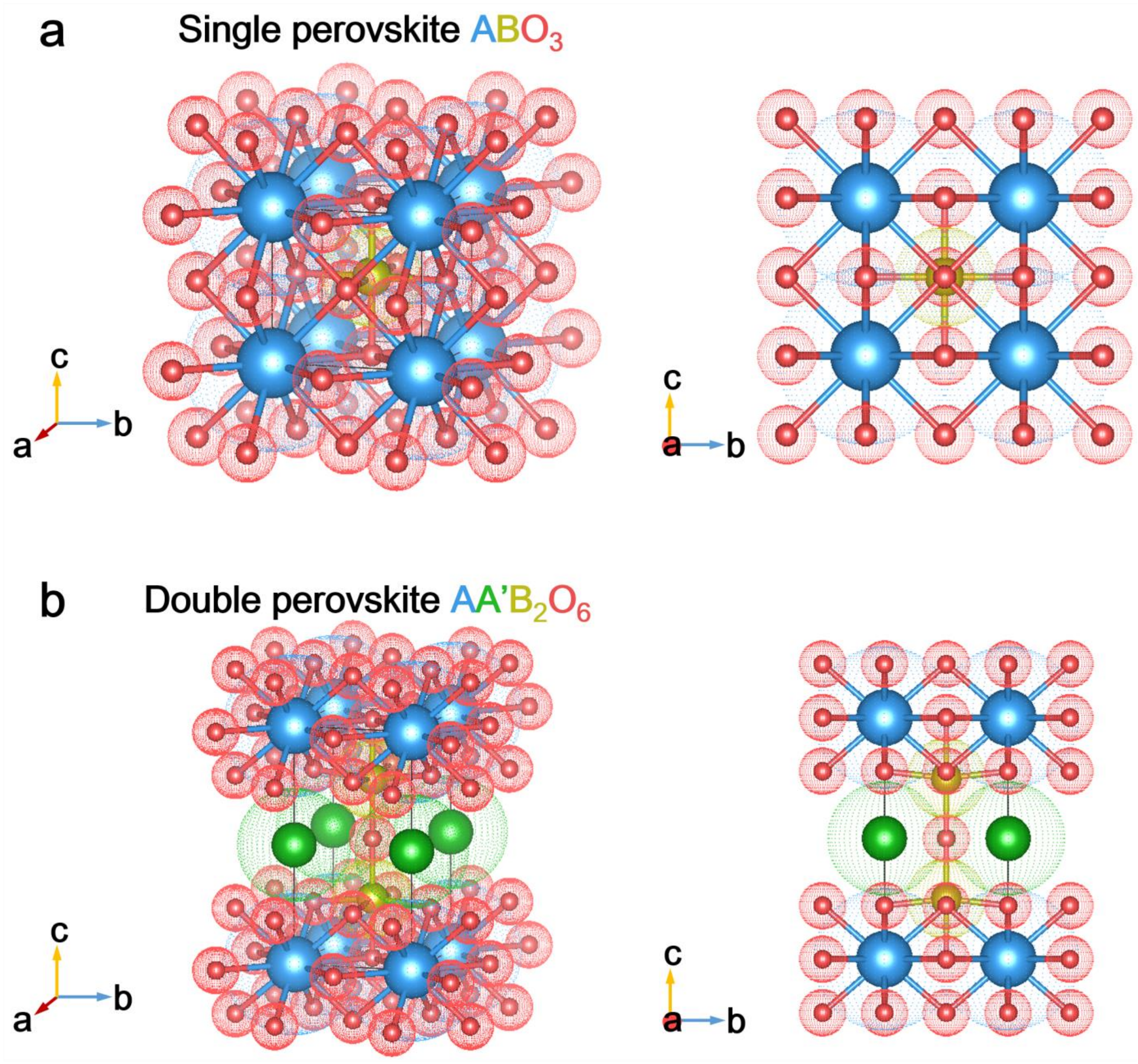

Supplementary Figure 10. Schematic description of the inductive effects and electron exchange interactions between A-sites and B-sites via electron clouds in **a,** single and **b,** double perovskites from molecular orbital theory.

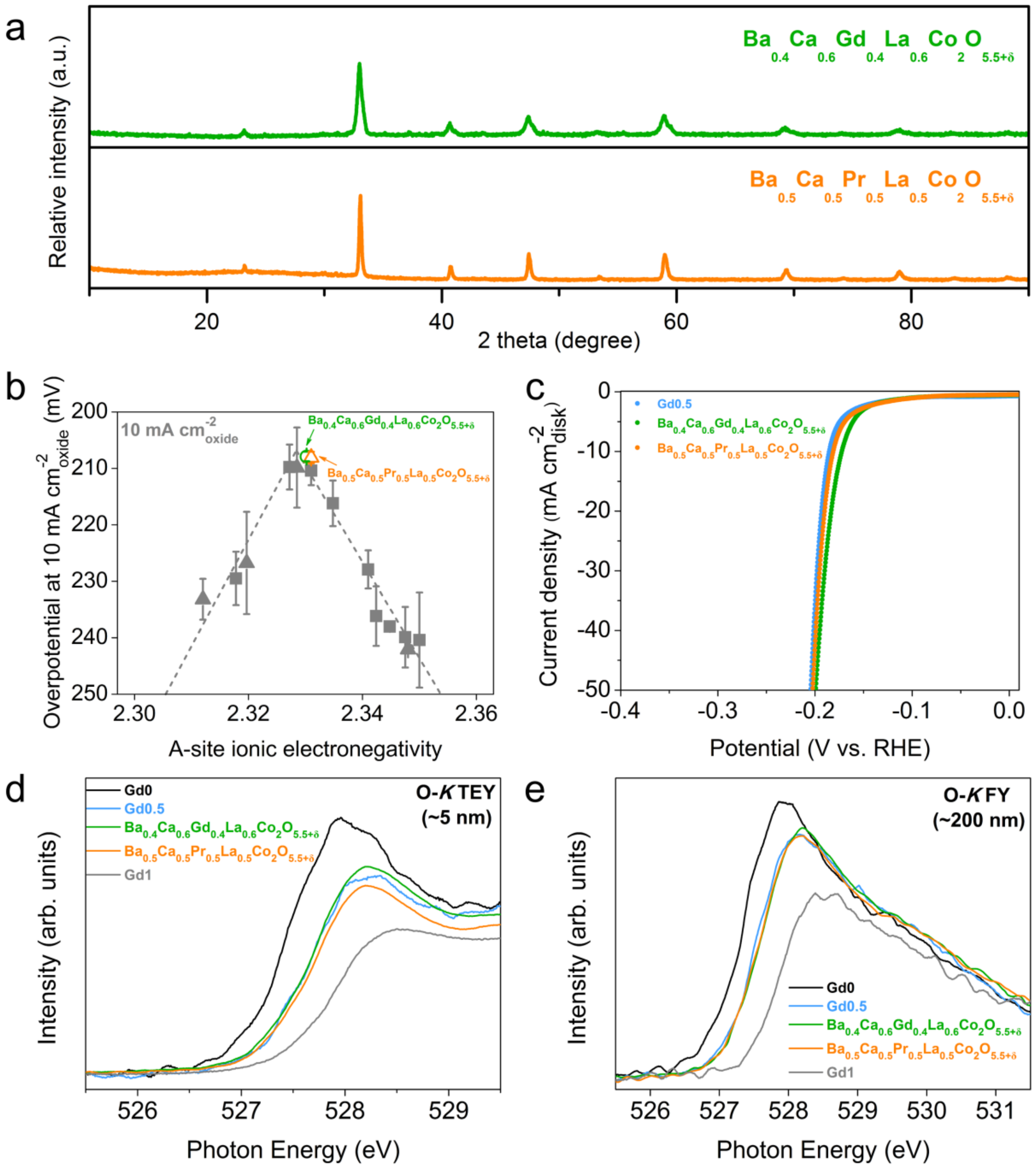

Supplementary Figure 11. Screening of perovskite candidates. a, XRD patterns of the new synthesized perovskites $Ba_{0.4}Ca_{0.6}Gd_{0.4}La_{0.6}Co_2O_{5.5+\delta}$ and $Ba_{0.5}Ca_{0.5}Pr_{0.5}La_{0.5}Co_2O_{5.5+\delta}$. **b,** HER activity trend of overpotential at 10 mA cm^{-2}_{oxide} as a function of A-site ionic electronegativity. **c,** Electrode HER polarization curves of Gd0.5, $Ba_{0.4}Ca_{0.6}Gd_{0.4}La_{0.6}Co_2O_{5.5+\delta}$ and $Ba_{0.5}Ca_{0.5}Pr_{0.5}La_{0.5}Co_2O_{5.5+\delta}$ catalysts. Soft XAS spectra at O-*K* edge collected in **d,** TEY and **e,** FY modes.

Supplementary Table 1. Calculated values of A-site ionic electronegativity, A-site ionic radius and A-O bond energy for all perovskites studied here.

Samples	A-site ionic electronegativity	A-site ionic radius (Å)	A-O bond energy (kJ mol^{-1})
Gd0	2.3120	2.970	-223.430
Gd0.5La0.4Sr0.1	2.3178	2.933	-216.766
Gd0.2	2.3196	2.952	-223.086
Gd0.4	2.3272	2.934	-222.742
Pr0.5La0.5	2.3285	2.950	-220.620
Gd0.5	2.3310	2.925	-222.570
Gd0.6	2.3348	2.916	-222.398
Sm0.5La0.5	2.3410	2.930	-214.695
Gd0.8	2.3424	2.898	-222.054
Ho0.8Ba0.6Sr0.6	2.3448	2.814	-206.986
Pr0.5Gd0.5	2.3475	2.905	-219.760
PrBa0.5Sr0.5	2.3480	2.845	-218.995
Gd1	2.3500	2.880	-221.710

Supplementary Table 2. BET specific surface areas and intrinsic HER activities of the catalysts.

Samples	Surface area (m^2 g^{-1})	η @ 1 mA cm^{-2}_{oxide} (mV)	η @ 10 mA cm^{-2}_{oxide} (mV)	Tafel slope (mV dec^{-1})
Gd0	2.836	193.6±3.9	233.2±3.6	42.5±0.4
Gd0.5La0.4Sr0.1	1.631	171.6±4.5	229.5±4.7	36.5±0.8
Gd0.2	3.255	183.4±2.8	226.8±9.1	40.6±1.1
Gd0.4	2.168	168.6±2.4	209.8±4.0	39.0±0.6
Pr0.5La0.5	2.337	174.5±8.3	209.9±7.1	35.5±1.6
Gd0.5	2.879	179.9±1.6	210.4±2.6	29.0±2.7
Gd0.6	2.035	182.7±3.5	216.2±4.0	31.1±0.4
Sm0.5La0.5	1.892	191.8±2.8	227.9±3.4	33.9±0.5
Gd0.8	2.071	197.1±2.3	236.2±5.3	32.9±1.6
Ho0.8Ba0.6Sr0.6	1.920	192.1±6.1	238.0±0.1	37.3±0.9
Pr0.5Gd0.5	3.263	198.1±5.8	239.9±5.4	34.2±1.1
PrBa0.5Sr0.5	2.694	204.9±0.6	242.1±0.7	36.7±0.2
Gd1	0.710	200.4±5.3	240.4±8.4	37.2±0.1

Supplementary Table 3. Results of XRD refinements for Gd0, Pr0.5La0.5, Gd0.5 and Gd1 perovskites.

Samples	Space group	Lattice parameter (Å)	χ^2	R_p (%)	R_{wp} (%)
Gd0	*Pm*-3*m*	*a*=3.893	3.055	2.970	4.130
Pr0.5La0.5	*Pm*-3*m*	*a*=3.885	4.490	4.410	6.530
Gd0.5	*P4/mmm*	*a*=7.801, *c*=7.644	1.670	2.730	3.700
Gd1	*Pmmm*	*a*=3.874, *b*=7.821, *c*=7.524	2.874	2.500	3.470

Supplementary Table 4. Comparisons of the disk current density at -240 mV and Tafel slope obtained on our best single and double perovskites with those of other well-known HER electrocatalysts in 1 M KOH solutions.

Catalysts	J_{disk}[a] (mA cm^{-2}_{disk}) @ -240 mV	Tafel slope (mV dec^{-1})	References
Gd0.5	338	27.6	This work
Pr0.5La0.5	317	33.8	This work
Pt/C	334	34.6	This work
Sr_2RuO_4	~160	51.0	Ref [2]
3% S-CoO NRs	~113	82.0	Ref [3]
L-0.5	~83	59.0	Ref [4]
A-PBCCF-H	~37	42.0	Ref [5]
CoP/CC	~19	129.0	Ref [6]
Ni/NC	~17	101.0	Ref [7]
Co@NG	~14	112.0	Ref [8]
Pr0.5BSCF	~13	45.0	Ref [1]
CoO_x@CN	~11	115.0	Ref [9]
Co/C	~3	134.0	Ref [10]

[a]The data of other electrocatalysts were achieved using the GETDATA software.

Supplementary Table 5. Comparisons of TOF values obtained on Gd0.5 material at different overpotentials with those of other state-of-the-art HER electrocatalysts.

Catalysts	TOF (H_2 s^{-1}) values at different overpotentials (V vs. RHE) in 1 M OH^-								Reference
	0.10 V	0.15 V	0.18 V	0.20 V	0.22 V	0.24 V	0.25 V	0.30 V	
Gd0.5	0.070	0.160	0.480	2.510	8.770	22.880	/	/	This work
Pt/C	0.370	0.700	0.970	1.170	1.410	1.710	/	/	This work
CoP_x	0.015	/	/	/	/	/	/	/	Ref [11]
$NiCo_2P_x$	0.056	/	/	/	/	/	/	/	Ref [11]
Ni_2P	/	/	/	0.014	/	/	/	/	Ref [12]
Ni-Mo	/	/	/	0.360	/	/	/	/	Ref [13]
γ-Mo_2N	/	/	/	/	/	/	0.070	/	Ref [14]
α-Mo_2C	/	/	/	/	/	/	2.500	/	Ref [14]
CoPO	/	/	/	/	/	/	/	2.620	Ref [15]
CoFePO	/	/	/	/	/	/	/	16.870	Ref [15]

Supplementary Note 1

Calculations of A-site parameters.

To save considerable time and costs in the design or choice of perovskites for alkaline HER, we seek to design a convenient, efficient and reliable A-site parameter with predictive power in perovskite lattice. A-site cations in perovskites generally possess 12-fold coordination with oxygen atoms, where alkaline-earth metal cations such as Ba^{2+} and Sr^{2+} are divalent and rare-earth ions such as lanthanides are trivalent[16-18]. Based on this condition, we calculate the average A-site ionic electronegativity, the average A-site ionic radius and the average A-site bond energy as follows:

(1) In this study, the average A-site ionic electronegativity (AIE) values are calculated according to the successful establishment of the new scale for ionic electronegativity by the Xue group[19,20], which was extracted from the basis of an effective ionic potential defined by the ionization energy and ionic radius. For Ba^{2+}, Sr^{2+}, La^{3+}, Pr^{3+}, Sm^{3+}, Gd^{3+} and Ho^{3+} with 12-fold coordination, the ionic electronegativity values are 1.087, 1.093, 1.225, 1.258, 1.283, 1.263 and 1.296 (Fig. 1a), respectively (detailed calculations can be found in the studies of the Xue group). Taking the calculation of the average A-site ionic electronegativity value for $(Gd_{0.5}La_{0.5})BaCo_2O_{5.5+\delta}$ (Gd0.5) as an example:

Average AIE (Gd0.5)

$= 0.5 \times AIE\ (Gd^{3+}) + 0.5 \times AIE\ (La^{3+}) + 1 \times AIE\ (Ba^{2+})$

$= 0.5 \times 1.263 + 0.5 \times 1.225 + 1 \times 1.087$

$= 2.331$

The average AIE values for the other perovskites are also calculated by the same procedure as Gd0.5.

(2) The average A-site ionic radius values are calculated based on the Shannon's and Jia's studies[21,22],

where the ionic radius values for Ba^{2+}, Sr^{2+}, La^{3+}, Pr^{3+}, Sm^{3+}, Gd^{3+} and Ho^{3+} with 12-fold coordination are 1.61, 1.44, 1.36, 1.32, 1.28, 1.27 and 1.23 Å (Fig. 1a), respectively. Also, taking the calculation of the average A-site ionic radius (AIR) value for $(Gd_{0.5}La_{0.5})BaCo_2O_{5.5+\delta}$ (Gd0.5) as an example:

Average AIR (Gd0.5)

= 0.5 × AIR (Gd^{3+}) + 0.5 × AIR (La^{3+}) + 1 × AIR (Ba^{2+})

= 0.5 × 1.27 + 0.5 × 1.36 + 1 × 1.61

= 2.925 Å

The average AIR values for the other perovskites are obtained by the identical calculation as Gd0.5.

(3) The average A-site bond energy values within the perovskites are estimated according to the prior works[23,24]:

$$\langle \text{A-O} \rangle = \Delta\,(\text{A-O}) + \Delta\,(\text{A'-O}) \tag{1}$$

$$\Delta\,(\text{A-O}) = \frac{X_A}{CN_A \cdot m} \times (\Delta H_{A_mO_n} - m \times \Delta H_A - \frac{n}{2} \times D_{O2}) \tag{2}$$

where X_A is the molar fraction of A-site metal; CN_A is 12; ΔH_{AmOn} and ΔH_A are the enthalpy of formation of one mole of A_mO_n oxide and the sublimation energy of A-site metal at 25 ℃ and D_{O2} is the dissociation energy of O_2 (500.2 kJ mol^{-1}). Taking the calculation of 1 molar fraction of Δ (Gd-O) as an example:

Δ (Gd-O)

$$= \frac{1}{12 \times 2} \times (\Delta H_{Gd2O3} - 2 \times \Delta H_{Gd} - \frac{3}{2} \times D_{O2})$$

$$= \frac{1}{12 \times 2} \times (-1819.6 - 2 \times 397.5 - \frac{3}{2} \times 500.2)$$

= -140.20 kJ mol^{-1}

Following the same calculation as Δ (Gd-O), the 1 molar fraction of Δ (Ba-O), Δ (Sr-O), Δ (La-O), Δ

(Pr-O), Δ (Sm-O) and Δ (Ho-O) are estimated as -81.51, -83.88, -141.92, -136.30, -124.45 and -134.69 kJ mol^{-1}, respectively. Then, taking the calculation of the average A-site bond energy (ABE) value for $(Gd_{0.5}La_{0.5})BaCo_2O_{5.5+\delta}$ (Gd0.5) as an example:

Average ABE (Gd0.5)

$= 0.5 \times \Delta (Gd\text{-}O) + 0.5 \times \Delta (La\text{-}O) + 1 \times \Delta (Ba\text{-}O)$

$= 0.5 \times (-140.20) + 0.5 \times (-141.92) + 1 \times (-81.51)$

$= -222.57$ kJ mol^{-1}

The average ABE values for the other perovskites are achieved by the identical calculation as Gd0.5.

Supplementary Note 2

Turnover frequency calculations.

In this work, TOF values are calculated according to a well-known method reported by the Jaramillo group[25,26]. Detailed process is shown as follows:

(1) To calculate the per-site TOF, we use the following formula:

$$\text{TOF}=\frac{\#\text{number of total hydrogen } turnovers/\text{cm}^2_{\text{geo}}}{\#\text{number of active sites/cm}^2_{\text{geo}}} \quad (3)$$

The total number of hydrogen turnovers is calculated from the current density according to:

$$\text{No. of H}_2 = \left(j\frac{\text{mA}}{\text{cm}^2_{\text{geo}}}\right)\left(\frac{1\ \text{C s}^{-1}}{1000\ \text{mA}}\right)\left(\frac{1\ \text{mol e}^{-1}}{96485.3\ \text{C}}\right)\left(\frac{1\ \text{mol H}_2}{2\ \text{mol e}^{-}}\right)\left(\frac{6.022\times10^{23}\ \text{H}_2\ \text{molecules}}{1\ \text{mol H}_2}\right)=3.12\times10^{15}\ \frac{\text{H}_2/\text{s}}{\text{cm}^2_{\text{geo}}}\ \text{per}\frac{\text{mA}}{\text{cm}^2_{\text{geo}}}$$

(2) We estimate the number of active sites as the number of surface metal sites, where Co and Pt atoms from respective unit cell of the Gd0.5 and Pt crystal structure are taken as possible active sites in line with previous works[27,28].

The active sites per real surface area are calculated from the following formula:

$$\text{No. of active sites} = \left(\frac{\text{No.of atoms/unit cell}}{\text{Volume/unit cell}}\right)^{\frac{2}{3}} \quad (4)$$

From the structures of Gd0.5 and Pt, we can calculate the number of active sites per real surface area for them:

$$\text{No. of active sites (Gd0.5)} = \left(\frac{8\ \text{atoms/unit cell}}{465.18\ \text{Å}^3\ \text{/unit cell}}\right)^{\frac{2}{3}} = 6.66\times10^{14}\ \text{atoms cm}^{-2}_{\text{real}}$$

$$\text{No. of active sites (Pt)} = \left(\frac{4\ \text{atoms/unit cell}}{61.35\ \text{Å}^3\ \text{/unit cell}}\right)^{\frac{2}{3}} = 1.62\times10^{15}\ \text{atoms cm}^{-2}_{\text{real}}$$

(3) The real surface area for HER is calculated from the electrochemical active surface area (ECSA), which can be converted from the specific capacitance. The specific capacitance for a flat surface is generally found to be in the range of 20-60 μF per cm^2_{geo}. In the following calculations of TOF we assume an average of 40 μF per cm^2_{geo} for Gd0.5 and Pt.

$$A_{ECSA} = \frac{\text{specific capacitance}}{40\ \mu F\ \text{per}\ cm^2_{geo}\ \text{per}\ cm^2_{ECSA}} \tag{5}$$

From Figure S8 we can calculate ECSA for Gd0.5 and Pt:

$$A_{ECSA}(Gd0.5)= \frac{2770\ \mu F\ \text{per}\ cm^2_{geo}}{40\ \mu F\ \text{per}\ cm^2_{geo}\ \text{per}\ cm^2_{ECSA}} =69.25\ cm^2_{ECSA}$$

$$A_{ECSA}(Pt)= \frac{15650\ \mu F\ \text{per}\ cm^2_{geo}}{40\ \mu F\ \text{per}\ cm^2_{geo}\ \text{per}\ cm^2_{ECSA}} =391.25\ cm^2_{ECSA}$$

(4) Finally, the plot of current density can be converted into a TOF plot according to the following formula:

$$TOF=\frac{(3.12\times10^{15}\ \frac{H2/s}{cm^2_{geo}}\ \text{per}\frac{mA}{cm^2_{geo}})\times|j|}{\text{No.of active sites} \times A_{ECSA}} \tag{6}$$

Supplementary Note 3

Explanations for the stability of Pt/C and perovskite materials.

In alkaline solutions, the HER comprises either Volmer-Heyrovsky or Volmer-Tafel pathways (Volmer step: $H_2O + e^- \rightarrow H_{ads} + OH^-$; Heyrovsky step: $H_{ads} + H_2O + e^- \rightarrow H_2 + OH^-$; Tafel step: $2H_{ads} \rightarrow H_2$)[29,30]. Tuning the adsorption/desorption of H_2O and hydrogen intermediates (H) for a catalyst plays a key role in the HER activity and stability[27,31]. Although the Pt materials are efficient for the adsorption/desorption of H intermediates, they are poor in the prior step of dissociating H_2O to H intermediates (Volmer step)[32]. Hence, the HER activity and stability of the Pt/C catalyst become worse because it cannot effectively cleave the H_2O to offer H intermediates for H_2 formation. Our bulk-phase perovskites with two active sites for both H_2O and H intermediates can maintain their HER activity and exhibit better durability[27].

Supplementary Note 4

Screening of new perovskite candidates.

Here, we further test the efficiency and reliability of our descriptor by screening new perovskite candidates in this system. Following our AIE design principle, we successfully synthesized two new cobalt-based perovskites with an AIE value of ~2.33 (namely $Ba_{0.4}Ca_{0.6}Gd_{0.4}La_{0.6}Co_2O_{5.5+\delta}$ and $Ba_{0.5}Ca_{0.5}Pr_{0.5}La_{0.5}Co_2O_{5.5+\delta}$ as shown in Supplementary Fig. 11a). According to our prior conclusions, cobalt-based perovskites with an AIE value of ~2.33 synthesized under the identical conditions in this system can be associated with the optimal electronic states of active B-sites via inductive effect in perovskite structure (including Co valence and Co-O bond covalency) and further show robust activity in alkaline HER. Therefore, the new synthesized perovskites are supposed to exhibit remarkable intrinsic and electrode HER activities. As expected, the intrinsic activity of the new synthesized perovskites can also climb to the top of the volcano plot (Supplementary Fig. 11b) and the electrode activity is comparable to Gd0.5 (Supplementary Fig. 11c). To further check whether the new perovskites possess the optimal electronic states of Co ions (such as Co valence and Co-O bond covalency) like Gd0.5, we performed the soft XAS spectra at the O-K edge collected by both TEY and FY modes, which are sensitive to the surface (~5 nm) and bulk (~200 nm) information, respectively. We also found that the electronic states of the new perovskites are similar to those of Gd0.5 (Supplementary Fig. 11d,e), demonstrating the high efficiency and reliability of our descriptor and strengthening our conclusions and results.

Supplementary References

1 Xu, X. et al. A perovskite electrocatalyst for efficient hydrogen evolution reaction. *Adv. Mater.* **28**, 6442-6448 (2016).

2 Zhu, Y. et al. Unusual synergistic effect in layered Ruddlesden-Popper oxide enables ultrafast hydrogen evolution. *Nat. Commun.* **10**, 149 (2019).

3 Ling, T. et al. Activating cobalt(II) oxide nanorods for efficient electrocatalysis by strain engineering. *Nat. Commun.* **8**, 1509 (2017).

4 Hua, B., Li, M., Zhang, Y. Q., Sun, Y. F. & Luo, J. L. All-in-one perovskite catalyst: smart controls of architecture and composition toward enhanced oxygen/hydrogen evolution reactions. *Adv. Energy Mater.* **7**, 1700666 (2017).

5 Hua, B. et al. A coupling for success: controlled growth of Co/CoO_x nanoshoots on perovskite mesoporous nanofibres as high-performance trifunctional electrocatalysts in alkaline condition. *Nano Energy* **32**, 247-254 (2017).

6 Tian, J., Liu, Q., Asiri, A. M. & Sun, X. Self-supported nanoporous cobalt phosphide nanowire arrays: an efficient 3D hydrogen-evolving cathode over the wide range of pH 0-14. *J. Am. Chem. Soc.* **136**, 7587-7590 (2014).

7 Zhang, X. et al. Facile synthesis of nickel-iron/nanocarbon hybrids as advanced electrocatalysts for efficient water splitting. *ACS Catal.* **6**, 580-588 (2015).

8 Zeng, M. et al. Metallic cobalt nanoparticles encapsulated in nitrogen-enriched graphene shells: its bifunctional electrocatalysis and application in zinc-air batteries. *Adv. Funct. Mater.* **26**, 4397-4404 (2016).

9 Jin, H. et al. In-situ cobalt-cobalt oxide/N-doped carbon hybrids as superior bifunctional electrocatalysts for hydrogen and oxygen evolution. *J. Am. Chem. Soc.* **137**, 2688-2694 (2015).

10 Liang, H. W. et al. Molecular metal-N_x centres in porous carbon for electrocatalytic hydrogen evolution. *Nat. Commun.* **6**, 7992 (2015).

11 Zhang, R. et al. Ternary $NiCo_2P_x$ nanowires as pH-universal electrocatalysts for highly efficient hydrogen evolution reaction. *Adv. Mater.* **29**, 1605502 (2017).

12 Laursen, A. B. et al. Nanocrystalline Ni_5P_4: a hydrogen evolution electrocatalyst of exceptional efficiency in both alkaline and acidic media. *Energy Environ. Sci.* **8**, 1027-1034 (2015).

13 McKone, J. R., Sadtler, B. F., Werlang, C. A., Lewis, N. S. & Gray, H. B. Ni-Mo nanopowders for efficient electrochemical hydrogen evolution. *ACS Catal.* **3**, 166-169 (2013).

14 Ma, L., Ting, L. R. L., Molinari, V., Giordano, C. & Yeo, B. S. Efficient hydrogen evolution reaction catalyzed by molybdenum carbide and molybdenum nitride nanocatalysts synthesized via the urea glass route. *J. Mater. Chem. A* **3**, 8361-8368 (2015).

15 Duan, J., Chen, S., Vasileff, A. & Qiao, S. Z. Anion and cation modulation in metal compounds for bifunctional overall water splitting. *ACS Nano* **10**, 8738-8745 (2016).

16 Hwang, J. et al. Perovskites in catalysis and electrocatalysis. *Science* **358**, 751-756 (2017).

17 Zhao, B. et al. A tailored double perovskite nanofiber catalyst enables ultrafast oxygen evolution. *Nat. Commun.* **8**, 14586 (2017).

18 Grimaud, A. et al. Double perovskites as a family of highly active catalysts for oxygen evolution

in alkaline solution. *Nat. Commun.* **4**, 2439 (2013).
19 Li, K. & Xue, D. Estimation of electronegativity values of elements in different valence states. *J. Phys. Chem. A* **110**, 11332-11337 (2006).
20 Li, K., Wang, X., Zhang, F. & Xue, D. Electronegativity identification of novel superhard materials. *Phys. Rev. Lett.* **100**, 235504 (2008).
21 Shannon, R. D. Revised effective ionic radii and systematic studies of interatomic distances in halides and chalcogenides. *Acta Cryst.* **32**, 751-767 (1976).
22 Jia, Y. Q. Crystal radii and effective ionic radii of the rare earth ions. *J. Solid State Chem.* **95**, 184-187 (1991).
23 Konysheva, E. Y., Xu, X. & Irvine, J. T. On the existence of A-site deficiency in perovskites and its relation to the electrochemical performance. *Adv. Mater.* **24**, 528-532 (2012).
24 Lide, D. R. *CRC Handbook of Chemistry and Physics, 90th ed.* (CD-ROM Version 2010) (CRC Press, 2009).
25 Kibsgaard, J. et al. Designing an improved transition metal phosphide catalyst for hydrogen evolution using experimental and theoretical trends. *Energy Environ. Sci.* **8**, 3022-3029 (2015).
26 Kibsgaard, J. & Jaramillo, T. F. Molybdenum phosphosulfide: an active, acid-stable, earth-abundant catalyst for the hydrogen evolution reaction. *Angew. Chem. Int. Ed.* **53**, 14433-14437 (2014).
27 Guan, D. et al. Searching general sufficient-and-necessary conditions for ultrafast hydrogen-evolving electrocatalysis. *Adv. Funct. Mater.* **29**, 1900704 (2019).
28 Mahmood, J. et al. An efficient and pH-universal ruthenium-based catalyst for the hydrogen evolution reaction. *Nat. Nanotechnol.* **12**, 441-446 (2017).
29 Jiao, Y., Zheng, Y., Jaroniec, M. & Qiao, S. Z. Design of electrocatalysts for oxygen- and hydrogen-involving energy conversion reactions. *Chem. Soc. Rev.* **44**, 2060-2086 (2015).
30 Gong, M. et al. Nanoscale nickel oxide/nickel heterostructures for active hydrogen evolution electrocatalysis. *Nat. Commun.* **5**, 4695 (2014).
31 Luo, Y. et al. Two-dimensional MoS_2 confined $Co(OH)_2$ electrocatalysts for hydrogen evolution in alkaline electrolytes. *ACS Nano* **12**, 4565-4573 (2018).
32 Subbaraman, R. et al. Enhancing hydrogen evolution activity in water splitting by tailoring Li^+-$Ni(OH)_2$-Pt interfaces. *Science* **334**, 1256-1260 (2011).
